# New Approach of UAV Movement Detection and Characterization Using Advanced Signal Processing Methods Based on UWB Sensing

**DOI:** 10.3390/s20205904

**Published:** 2020-10-19

**Authors:** Angela Digulescu, Cristina Despina-Stoian, Denis Stănescu, Florin Popescu, Florin Enache, Cornel Ioana, Emanuel Rădoi, Iulian Rîncu, Alexandru Șerbănescu

**Affiliations:** 1 Telecommunications and Information Technology Department, Military Technical Academy “Ferdinand I”, 050141 Bucharest, Romania; cristina.despina@mta.ro (C.D.-S.); denis.stanescu@mta.ro (D.S.); florin.popescu@mta.ro (F.P.); florin.enache@mta.ro (F.E.); iulian.rincu@mta.ro (I.R.); alexandru.serbanescu@mta.ro (A.Ș.); 2 GIPSA-Lab, Université Grenoble Alpes, 38400 Saint Martin d’Hères, France; cornel.ioana@gipsa-lab.grenoble-inp.fr; 3 Lab-STICC, CNRS, UMR 6285, Université de Bretagne Occidentale, 29200 Brest, France; emanuel.radoi@univ-brest.fr

**Keywords:** drone, LSS UAV, UWB sensing, signal processing, movement map, RPA

## Abstract

In the last years, the commercial drone/unmanned aerial vehicles market has grown due to their technological performances (provided by the multiple onboard available sensors), low price, and ease of use. Being very attractive for an increasing number of applications, their presence represents a major issue for public or classified areas with a special status, because of the rising number of incidents. Our paper proposes a new approach for the drone movement detection and characterization based on the ultra-wide band (UWB) sensing system and advanced signal processing methods. This approach characterizes the movement of the drone using classical methods such as correlation, envelope detection, time-scale analysis, but also a new method, the recurrence plot analysis. The obtained results are compared in terms of movement map accuracy and required computation time in order to offer a future starting point for the drone intrusion detection.

## 1. Introduction

Considering their variety and the boundless applications in which they can be involved, the research interest in the drone field has grown exponentially in recent years. Nowadays, drones, also known as unmanned aerial vehicles (UAV), are widely used in both civilian and military environments representing an important data source for inspection, surveillance, mapping, and modeling applications [1,2,3,4,5]. 

The drones increasing cost-effectiveness, along with their improved capabilities, has also led to their transformation into a threat. Being suitable for offensive and intelligence, surveillance, and reconnaissance (ISR) applications, the attackers pay most attention to the low, slow and small UAV (LSS UAV) category that is difficult to detect [6]. The US Federal Aviation Administration publishes periodically reports of unmanned aircraft systems (UAS) and a dramatic increase of worldwide drone-related incidents and intrusions can be observed [7]. Consequently, the drone detection represents a topical issue and a key element in ensuring security for public and classified areas. 

The state-of-the-art solutions on drone detection are classified in the function of technologies involved: Visual, acoustic, radio-frequency (RF), and multimodal detection [8]. 

Drone detection using images or videos captured by camera sensors is based on computer vision algorithms and can be performed by traditional machine learning algorithms [9,10] or deep learning-based algorithms [11,12,13]. For this approach, the line-of-sight (LoS) is mandatory and the performance depends on the quality of the image captured that may be degraded by adverse environment conditions such as fog, cloud, dust, or low ambient light. The detection results can be improved using a thermal, laser-based, or panoramic camera involving a much higher cost. 

Employing the sound produced by a flying drone, the acoustic detection eliminates the possibility of confusing birds or planes with drones [14], which represents an alternative for the visual approaches. The audio method is effective only at very short distances; thus, the authors in [15] test the effectiveness of the sound classification methods (Gaussian Mixture Model, Convolutional Neural Network, and Recurrent Neural Network) using mel-frequency cepstrum coefficients (MFCC) and mel-spectrogram features and state that after 150 m the results are irrelevant. The noisy environment represents another challenge for this method. Therefore, the approaches from [16,17] increase the robustness against noise proposing advanced acoustic feature extracting techniques. To achieve better performance, the research community uses hybrid solutions that process huge data sets acquired by large arrays of spatially-distributed acoustic and visual sensors [18,19]. 

Considering the increased robustness to weather and illumination conditions, the RF drone detection methods are widely used because they can ensure a larger coverage area than the previous approaches. The RF signals related techniques are classified in active (involving radar sensors) and passive surveillance techniques. Typically, between drones and their controller exists the uplink and downlink transmissions that use the industrial, scientific, and medical (ISM) frequency spectrum. The same bandwidth is used by an enormous number of Wi-Fi and Bluetooth devices that can interfere and cover the target transmission. Based on the bandwidth and modulation feature of the RF detected signal, in [20] it is used, in the first stage of the drone classification algorithm, a two-state Markov model that decides if the signal comes from a drone controller or an interference source. Other approaches aim to minimize the complexity of the algorithms involved in drone passive detection and propose new specialized methods depending on the physical layer protocol used by the drone [21,22]. Currently, the drone industry provides autonomous guidance capabilities, based on global positioning system (GPS), and makes drones flying in fully autonomous mode, impossible to be detected by RF passive techniques.

The surveillance systems based on radar sensors represent a reliable solution for autonomous UAV detection. Considering the LLS UAV dimensions and the fact that they are mostly made of plastic, the radar cross-section is very small. The new approaches try to improve the detection capabilities using enhanced solutions based on the multi-static radar system [23], empirical mode decomposition [24], backscattering from rotating parts such as propellers and rotors to evaluate micro-doppler signatures [25], or different approaches to the frequency modulated continuous wave radar [26,27]. For a recent and comprehensive review of the radar based detection techniques, the reader is referred to [28].

Considering the LSS UAV intrusion challenges and the different methods previously presented, this paper proposes a new approach for the UAV/drone movement detection and characterization based on ultra-wide band (UWB) sensing. This sensing system is composed of two UWB sensors and uses different signal processing methods in order to obtain an accurate detection and characterization of the movement. The experiments are carried out indoor, but the presented approach can also be extended for outdoor situations.

To the best of our knowledge, no UWB sensing approach has been proposed so far for drones intrusion detection, but only for their pose estimation in cooperative mode [29,30]. Considering the accuracy provided by the UWB technology, reference [31] presents the results achieved for pedestrian indoor localization and reference [32] tries to characterize and detect the person fall.

Our approach has the big advantage to sense a small dimension moving target built from heterogeneous materials (plastic and metal) which makes it a suitable candidate for the detection of such COTS-commercial of the shelf UAVs. Another advantage is the UWB technology capability to detect the movement even in non-line-of-sight [33] scenarios compared with visual-based technologies. Moreover, the UWB technology has the advantage to use a different frequency bandwidth than the previously mentioned RF technologies, which limits the ubiquitous ISM interferences. Hence, the determinant factors of the choice of the UWB technology are its advantages such as movement detection, low power, and affordable price [33].

The paper is organized as follows: Section 2 presents the experimental setup and the measurement configurations. In Section 3, we describe the signal processing methods used for the drone movement detection and characterization, emphasizing the recurrence plots analysis (RPA) method. In Section 4, we illustrate the results obtained for each configuration with the proposed analysis methods and we discuss the performance of each method. Finally, Section 5 presents the conclusions and further developments of our work.

## 2. Experimental Setup

**Figure 1:**
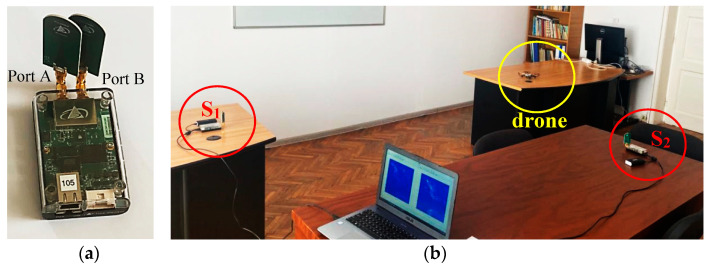
(**a**) The PulsON 440 ultra-wide band (UWB) sensor used for drone movement characterization, (**b**) the sensor placement for the experimental setup.

The experiment was performed using two PulsON 440 UWB sensors [34] (Figure 1a) connected to a laptop, as presented in Figure 1b. The sensors, S_1_ and S_2_, are placed at the same height. The choice of this minimum number of sensors is justified by the fact that the drone movement is carried out in a horizontal or vertical plane. 

**Figure 2:**
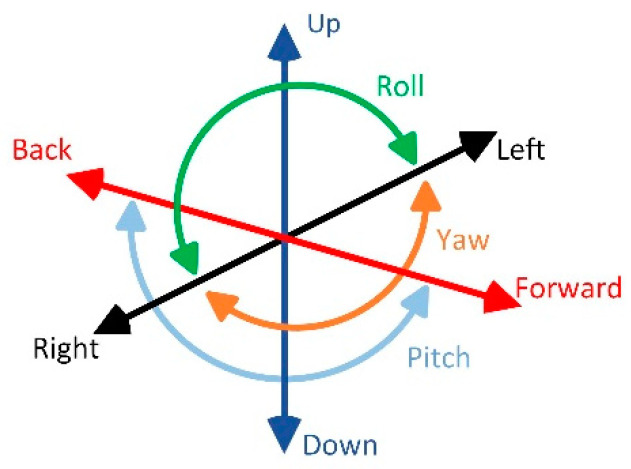
Drone possible movement scenarios.

Each measurement is carried out for approximately 40 s performing a total of 400 scan lines in three different configurations (the reference is the sensor S_1_): Left-right movement, up-down movement, and forward-back movement (Figure 2).

**Figure 3:**
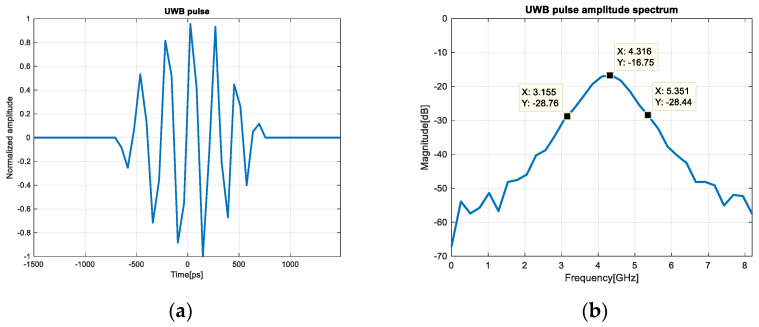
(**a**) Emitted signal, (**b**) amplitude spectrum of the emitted signal.

The sensors are configured in a mono-static radar configuration with the highest transmit gain [34] and for both ports, A and B, the standard time domain BroadSpec antennas [35] were connected. The emitted sensors signals (Figure 3) are wide-band pulses with a bandwidth of (3.1 and 5.3 GHz) and the maximum transmit power spectral density of −41 dBm/MHz [34]. 

**Figure 4:**
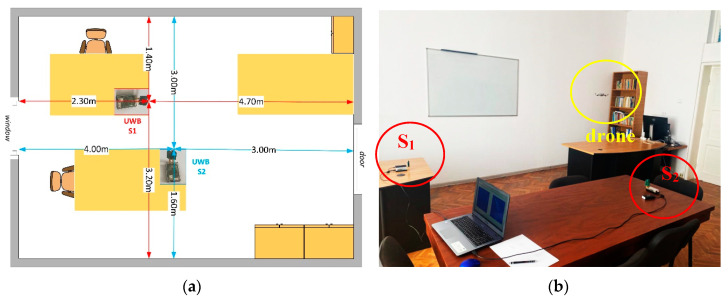
(**a**) The UWB sensors placement, (**b**) experimental positioning of the drone and UWB sensors.

The placement of the UWB sensors is shown in Figure 4. In the room, there are three tables with the same height (80 cm) and three cabinets along the wall accordingly, as shown in Figure 4. The height of the room is 2.5 m. The drone was controlled above the tables.

**Figure 5:**
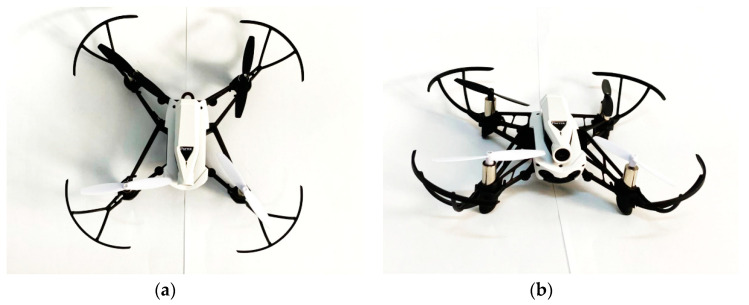
(**a**) The Parrot Mambo FPV drone-top view, (**b**) the Parrot Mambo FPV drone-frontal view.

The drone Parrot Mambo FPV used for the experiment in Figure 5 is equipped with four rotors, with a weight of 63 g and the dimensions 18 × 18 × 4 cm^3^ [36]. The device was controlled with a dedicated smartphone application via Bluetooth which, given the frequency band of the technology, did not interfere with the UWB sensors. 

Due to its weight and dimensions, the drone is classified as micro UAV [37]. Drones with these dimensions present several limitations such as the maximum time flight or the maximum range, but because of their very small dimensions, they are the most difficult to be detected or for their movement to be characterized. 

## 3. Advanced Signal Processing Methods

In order to detect the drone movement and characterize it, several methods of signal analysis were used: Classical methods such as correlation, envelope detection, spectrogram, or wavelet transform, respectively more recent methods, such as the recurrence plot analysis (RPA).

### 3.1. Correlation

Correlation is a linear signal processing method that measures the degree of similarity of two signals when no other different parameters are known, such as relative position or phase change. When the two signals overlap, the correlation is maximum and it decreases as one signal is translated from the other with the time shifting lag τ. 

When the translation is large enough and no similar patterns are found in the signal, no correlation can be found and its value becomes almost zero. If repetitive patterns are found in the signal, the correlation function provides secondary maxima [38].

In this experiment, the correlation is calculated between the difference of each successive scan lines and the emitted signal from Figure 3. This leads to the following equation for the expression of correlation:

(1) $$r_{i}\left(\tau \right)=\frac{1}{T}\int_{0}^{T}e\left(t\right)\cdot d_{i}\left(t+\tau \right)dt,i=1,2,\ldots ,399$$

where e(t) is the emitted signal and

(2) $$d_{i}(t)=s_{i}\left(t\right)-s_{i+1}\left(t\right),i=1,2,\ldots ,399$$

and $s_{i}\left(t\right)$ is the $i^{th}$ scan line.

The correlation movement map is represented by performing the difference between each two successive correlation operations according to the equation:

(3) $$MM_{c}=\left[\begin{array}{c} r_{1}\left(t\right)\\ r_{2}\left(t\right)\\ \vdots \\ r_{399}\left(t\right) \end{array}\right]$$

The analysis based on the correlation is often used in the time series analysis and signal processing, and is very useful in applications where there are similar signals present. Its main advantage is that it is the optimal detector of a signal embedded in noise [38].

### 3.2. Envelope Detection

In the field of radio communications, one of the elementary mathematical notions is given by the analytical signal. To obtain this signal, the Hilbert transform is applied to a real signal d(t). The real part of the analytical signal is given by the signal on which the transformation is applied and the imaginary part is actually the Hilbert transform of the signal. Moreover, the magnitude of the analytical signal represents the complex envelope of the real signal [39].

In order to detect the envelope, the Hilbert transform is applied on the result obtained from the difference between each two successive scan lines, $d_{i}(t)$ (Equation (2)), using the formula:

(4) $$h_{i}\left(t\right)=H\left\{d_{i}\left(t\right)\right\}=\frac{1}{\pi }\int_{-\infty }^{\infty }\frac{d_{i}\left(\tau \right)}{t-\tau }d\tau =d_{i}\left(t\right)*\frac{1}{\pi t},i=1,2,\ldots ,399$$

As it can be seen, $h_{i}(t)$ is a linear function and can be written as the convolution of the real signal $d_{i}\left(t\right)$ with the function $\frac{1}{\pi t}$ [39].

Using this information, we conclude that the analytical signal has the following expression:

(5) $$z_{i}\left(t\right)=d_{i}\left(t\right)+jh_{i}\left(t\right),i=1,2,\ldots ,399$$

The envelope detection involves creating the analytic signal by using the Hilbert transform. Mathematically, it is defined as [40]:

(6) $$env_{i}\left(t\right)=\sqrt{d_{i}{\left(t\right)}^{2}+h_{i}{\left(t\right)}^{2}},i=1,2,\ldots ,399$$

The envelope detection movement map is given by the equation:

(7) $$MM_{env}=\left[\begin{array}{c} env_{1}\left(t\right)\\ env_{2}\left(t\right)\\ \vdots \\ env_{399}\left(t\right) \end{array}\right]$$

This approach has the advantage to demand low computational resources, but it is noise sensitive.

### 3.3. Spectrogram

One of the most used methods in the field of signal detection consists of the linear transformation of signals from the time domain into the frequency domain to measure the instantaneous frequency of the studied signal. Hereby, the short-time Fourier transform (STFT) is applied on a time sliding window g(t) of the analyzed signal $d_{i}(t)$ (Equation (2)), which is the difference between each two successive scan lines [41]:

(8) $$S_{i}\left(u,f\right)=\int_{-\infty }^{\infty }d_{i}\left(t\right)\cdot g\left(t-u\right)\cdot e^{-j2\pi ut}dt,\;i=1,2,\ldots ,399$$

STFT visualization is often performed using the spectrogram, which defines the energy density and measures the signal energy in a time-frequency cell, which renders its resolution [41].

(9) $$P_{i}\left(u,f\right)={\left|S_{i}\left(u,f\right)\right|}^{2}={\left|\int_{-\infty }^{\infty }d_{i}\left(t\right)\cdot g\left(t-u\right)\cdot e^{-j2\pi ut}dt\right|}^{2},\;i=1,2,\ldots ,399$$

The length of the window affects the time-frequency resolution. A narrow window results in a fine resolution in time, but a rough resolution in frequency and a wide window results in a fine resolution in frequency and rough resolution in time. This compromise between time and frequency is the main disadvantage of this method [41].

The spectrogram movement map is given by the equation:

(10) $$MM_{s}=\left[\begin{array}{c} \sum_{f}P_{1}\left(u,f\right)\\ \sum_{f}P_{2}\left(u,f\right)\\ \vdots \\ \sum_{f}P_{399}\left(u,f\right) \end{array}\right]$$

Combining time and frequency domain analysis, the spectrogram is a powerful tool used in detection applications in noisy environments. It provides the time information of the frequency components in a time-varying signal, but it is limited by the time-frequency resolution.

### 3.4. Wavelet Transform

The wavelet transform is a linear transformation, similar to the Fourier transform, which has a higher accuracy than the previous one when it comes to locating in time the different frequency components of a signal, this being widely used as an efficient analysis tool. In the field of signal analysis, one of the most notable functions is the wavelet function, a mathematical function of zero mean [42]:

(11) $$\int_{-\infty }^{\infty }\psi_{0}\left(t\right)dt=0$$

On this foundation, the idea that proposes the creation of an orthonormal base elaborated from this wavelet and from its dilated and delayed variants is created. In this process, the wavelet is dilated with the scale parameter s and translated with the parameter τ.

(12) $$\psi_{\tau ,s}\left(t\right)=\frac{1}{\sqrt{s}}\psi \left(\frac{t-\tau }{s}\right)$$

Considering the difference between each two successive scan lines $d_{i}(t)$ (Equation (2)) as an input parameter, the wavelet analysis is performed [42].

(13) $$W_{i}\left(\tau ,s\right)=\frac{1}{\sqrt{s}}\int_{-\infty }^{\infty }d_{i}\left(t\right)\cdot \psi^{*}\left(\frac{t-\tau }{s}\right)dt,\;i=1,2,\ldots ,399$$

As a result of this design, we obtain a two-dimensional function called a scalogram which is able to quantify the time-frequency variations, but it must take into account the scale provided by the wavelet basis.

The movement map is given by the equation:

(14) $$MM_{w}=\left[\begin{array}{c} \sum_{s}W_{1}\left(\tau ,s\right)\\ \sum_{s}W_{2}\left(\tau ,s\right)\\ \vdots \\ \sum_{s}W_{399}\left(\tau ,s\right) \end{array}\right]$$

This method requires the definition of an appropriate wavelet dictionary so that it resembles as much as possible the analyzed signal x(t), therefore it must have a sufficient number of points to render the relevant features of the analyzed signal. In this case, the wavelet transform has the advantage of an analysis in which adjustable windows are used, being more advantageous than the fixed ones specific to the short-time Fourier transform.

### 3.5. Recurrence Plot Analysis

This method is based on extracting dynamic information about the sequence of values of an input data set, being an analysis of a nonlinear time series. Its foundation consists of the construction of the phase space of the analyzed system from which the data are extracted. A phase space is a space in which all possible states of a system are represented, each possible state corresponding to a unique point in the phase space.

Starting from the difference between each two successive scan lines $d_{i}(t)$ (Equation (2)) that is considered as a time series [43,44], the construction of the phase space is given by the transition from the initial values of the time series to a vector that defines the new representation space. This vector is obtained by introducing two new parameters: Time delay d and phase space dimension m. Thus, the equation that defines the new vector is [43,44]:

(15) $$\vec{v_{j}}=\sum_{k=1}^{m}d_{i}\left[j+\left(k-1\right)d\right]\cdot \vec{e_{k}},\;i=1,2,\ldots ,399$$

where $\vec{e_{k}}$ are the axis vector units. The choice of the parameters m and d can be found in [44].

**Figure 6:**
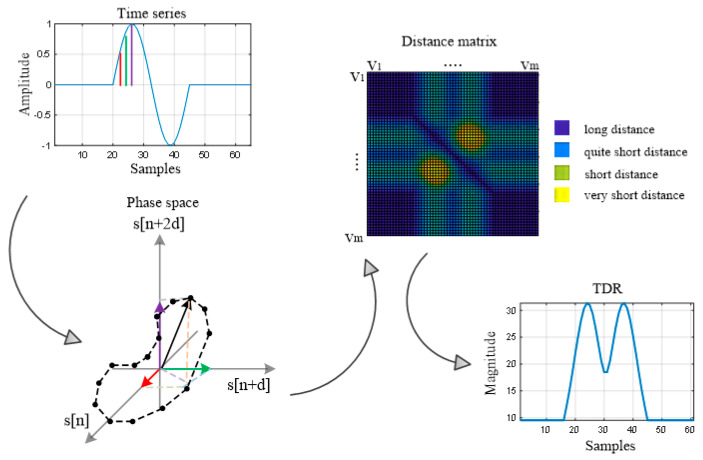
The illustration of the recurrence plots analysis (RPA) method.

This method (Figure 6) allows investigating the trajectory of the *m*-dimensional phase space through a two-dimensional representation of the pair-wise distance, a representation called the recurrence plot. Mathematically, this is expressed as the distance matrix, a form of representation of the distances between all pair-wise trajectory points and provides a two-dimensional alternative to represent the trajectory [43,44]:

(16) $$D_{j,k}=\left\|\vec{v_{j}}-\vec{v_{k}}\right\|,j,k=\left\{1,2,3,\ldots ,M\right\}$$

where M=N−(m−1)d, N is the number of samples of the signal $d_{i}\left(t\right)$ and in our paper, ‖·‖ is the Euclidian distance. For this application, we chose the Euclidean distance, because our purpose is to best highlight sudden changes in the phase space trajectory, these changes corresponding to the UWB pulses of the movement [43].

Next, the time-distributed recurrence (TDR) is defined in order to highlight the sudden changes in the trajectory [43]:

(17) $$TDR_{i}=\sum_{j=1}^{M}D_{j,k},\;i=1,2,\ldots ,399$$

The TDR RPA movement map is given by the equation:

(18) $$MM_{TDR}=\left[\begin{array}{c} TDR_{1}\\ TDR_{2}\\ \vdots \\ TDR_{399} \end{array}\right]$$

The advantage of this method is that it is a non-parametric method and it can be analyzed for many types of signals [43,44]. 

## 4. Results and Discussion

In this section, the results obtained (using the methods described in Section 3) for each configuration (mentioned in Section 2) are presented. The movement maps representations are translated into range (in meters) instead of time (in seconds).

### 4.1. Forward-Back Movement

**Figure 7:**
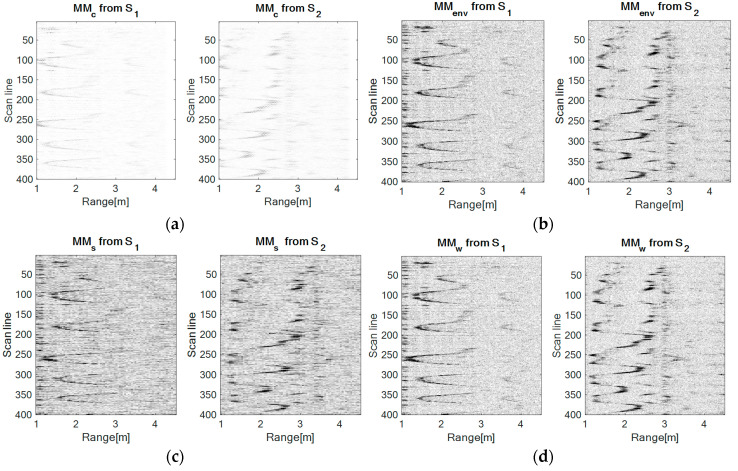

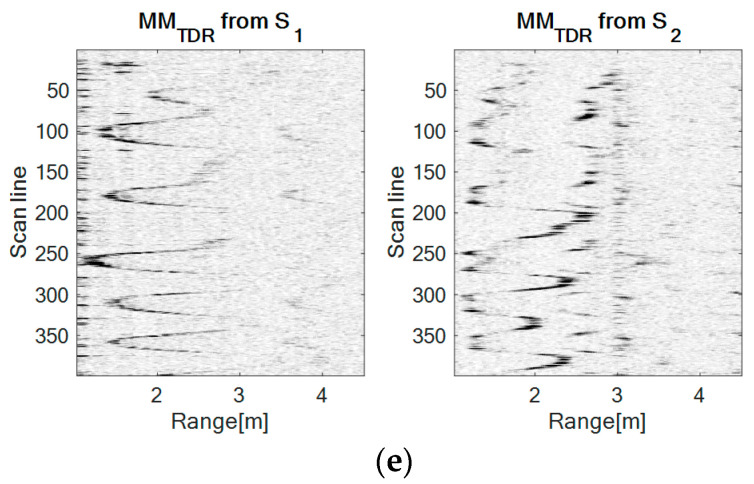
The movement maps for the forward-back configuration obtained with each method: (**a**) The correlation method, (**b**) the envelope detection method, (**c**) the spectrogram method: Window size: 64, number of overlapped samples: 60, number of FFT (Fast Fourier Transform) points: 128, (**d**) the wavelet transform method: Mother Morlet wavelet, (**e**) the time-distributed recurrence (TDR) RPA method: m = 5, d = 1.

In this configuration, the movement is repeatedly performed in front of the sensor S_1_ in the range 1–2.8 m, at a height of approximately 1.8 m. Hereby, the movement of the drone relative to the sensor S_2_ is a left-right movement. Figure 7 presents the results obtained with each method.

From Figure 7, it can be observed that the UWB sensor S_1_ senses the forward-back movement through a spike-motion, whereas the UWB sensor S_2_ perceives this movement as an oscillatory motion.

At a visual comparison level between the approached methods, the worst results are obtained using the correlation, next the spectrogram has better results, but it seems that the signals are very noisy. In fact, this representation is worsened by the time-frequency resolution. The envelope detection and the wavelet transform provide better results than the latter mentioned methods, but the TDR RPA approach delivers the best results, the salt and pepper noise effect (spots with minimum and maximum values of light intensity, producing randomly black and white pixels overlaid on the original image) being significantly reduced.

### 4.2. Up-Down Movement

**Figure 8:**
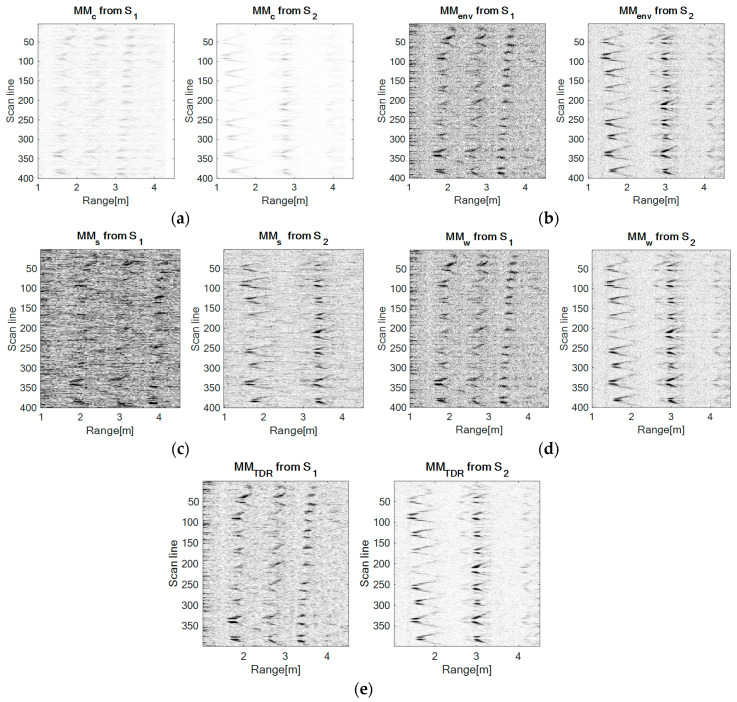
The movement maps for the up-down configuration obtained with each method: (**a**) The correlation method, (**b**) the envelope detection method, (**c**) the spectrogram method: Window size: 64, number of overlapped samples: 60, number of FFT points: 128, (**d**) the wavelet transform method: Mother Morlet wavelet, (**e**) the TDR RPA method: m = 5, d = 1.

In this configuration, the movement is repeatedly performed at a distance of 2.2 m from sensor S_1_ and a distance of 1.6 m from sensor S_2_, at a varying height range. Hereby, the movement of the drone relative to both sensors is similar. Figure 8 presents the results obtained with each method.

Relative to both sensors, the movement is similar, therefore, as expected, the movement maps corresponding to both sensors resemble a lot. The major difference between the recordings for each sensor is that sensor S_1_ acquires the signals in a higher proportion, given the different distances from the drone to the sensors. The motion is also oscillatory, but less deep than in the case of the forward-back configuration relative to the sensor S_2_. 

Visually, the performances of each method remain classified in the same order as in Section 4.1.

### 4.3. Left-Right Movement

In this case, the movement is performed at a distance of 2.5 m from sensor S_1_, but on the direction of sensor S_2_ in a forward-back movement. The movement of the drone relative to the sensor S_1_ is a left-right movement. Still, during the experiment the movement of the drone is not purely left-right relative to sensor S_1_, it was combined with a forward movement. Figure 8 presents the results obtained with each method.

**Figure 9:**
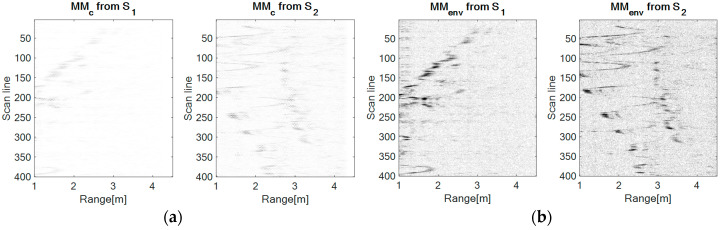

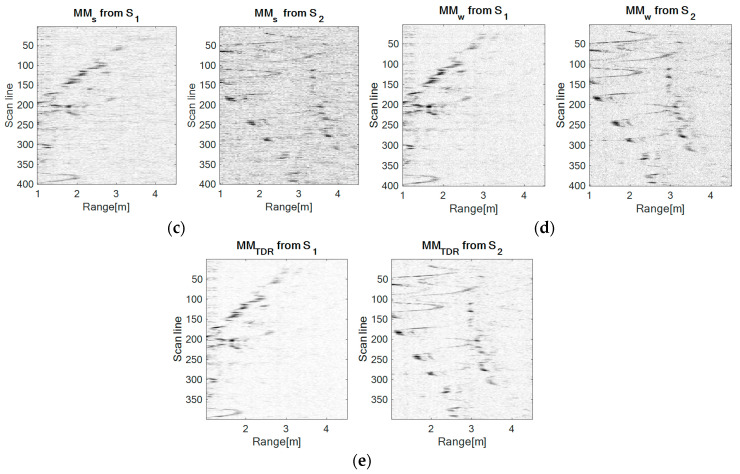
The movement maps for the left-right configuration obtained with each method: (**a**) The correlation method, (**b**) the envelope detection method, (**c**) the spectrogram method: Window size: 64, number of overlapped samples: 60, number of FFT points: 128, (**d**) the wavelet transform method: Mother Morlet wavelet, (**e**) the TDR RPA method: m = 5, d = 1.

Figure 9 shows that the UWB sensor S_2_ senses the forward-back movement through spike-motion which reduces their amplitude meaning that the drone distances from the sensor, whereas the UWB sensor S_1_ perceives this movement as an oscillatory motion (scan lines 150–200 and 350–399 which gets closer to sensor S_1_, confirming the forward component of the movement.

At a visual comparison level between the approached methods, the performances of each method remain classified in the same order as in Section 4.1

### 4.4. Discussion

The choice of the indoor LSS UAV movement detection and characterization is justified by the fact that such devices represent a potential threat for indoor/office intrusion for unauthorized surveillance/spying purposes. This paper presents a new technological solution for the LSS UAV movement detection that is characterized with different signal processing methods in UWB bandwidth.

This discussion regards a comparison between the first three methods from a performance point of view: Envelope detection, wavelet transform, and TDR RPA. The performance criteria considered for these methods are the image improvement and the computation time. 

The first criterion considers the mean of the differences between the movement maps of each corresponding method:

(19) $$MM\_acc=\frac{1}{l_{x}\cdot l_{y}}\cdot \left[e^{T}\cdot \left(\left|MM_{env}-MM_{test}\right|\right)\cdot e\right]$$

where e is a column vector whose all entries have value 1, $MM_{test}$ is the movement map under test (obtained with the wavelet transform or TDR RPA method), $l_{x}$ and $l_{y}$ are the pixel dimensions of the compared images (both images have the same dimensions). The fewer movement maps differences there are, the smaller the MM_acc parameter value is [45]. 

The second criterion determines the computation time on the same processing system and for the same measurement (the 400 scan lines from each configuration).

As presented in Table 1, although the envelope detection method is the most used in such types of applications with satisfactory results and shortest computation time, its limitation is given by the fact that it is noise sensitive.

The wavelet transform provides similar results with the envelope detection method. It needs an appropriate dictionary, otherwise its performance can be limited. Moreover, the computation time is higher than the envelope detection, but still usable for on-site applications.

**Figure 10:**
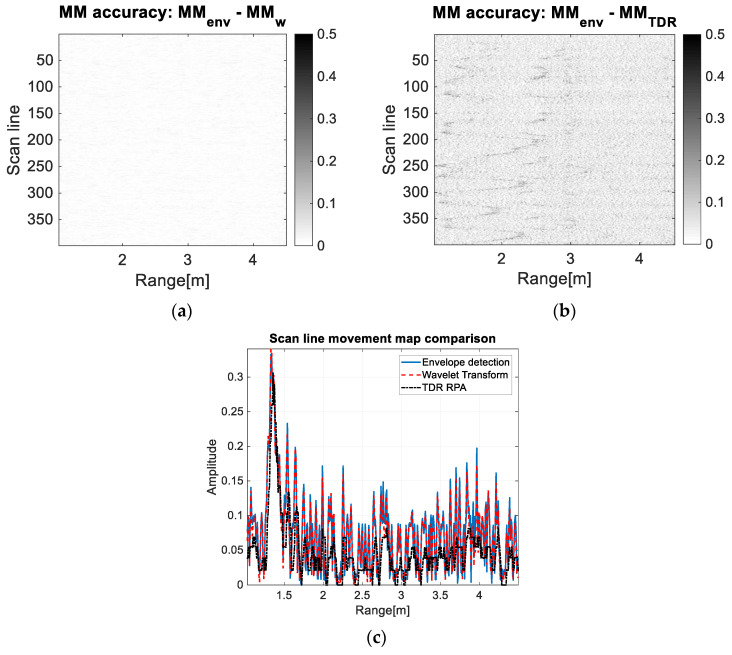
The forward-back movement maps accuracy. (**a**) The subtraction of the envelope detection map and the wavelet transform map: The maps are almost identical, (**b**) the subtraction of the envelope detection map and the TDR RPA map: The envelope detection map contains more salt-and-pepper noise, (**c**) the 100th scan line of each corresponding movement map.

From Figure 10a, it can be observed that the difference between the envelope detection movement map and the wavelet transform movement map contains almost unobservable noise and, also, the drone trajectory does not exist.

Figure 10b represents the difference between the envelope detection movement map and the TDR RPA movement map. It contains a visible salt-and-pepper effect because of the noise from the envelope detection movement map meaning that the noise effect is significantly reduced for the TDR RPA method. Moreover, in this figure, the LSS UAV trajectory is still visible implying that the trajectory is better emphasized by the TDR RPA approach.

Figure 10c presents the 100th scan line from the forward-back movement processed with the discussed methods. After computing the noise level (Appendix A) of each processed scan line, we obtained the following results: −22.3 dB for the envelope detection method, −22.7 dB for the wavelet transform method, respectively −27.1 dB for the TDR RPA method. Hereby, the noise level is approximately the same for the envelope detection method and for the wavelet transform method, whereas the TDR RPA method decreases the noise level with almost 5 dB. Moreover, the amplitude of the detected UWB pulse has approximately the same value (−15 dB for the envelope detection and wavelet transform methods, respectively −16 dB for the TDR RPA method). 

The time distributed recurrence based on the recurrence plot analysis (TDR RPA) method mostly improves the quality of the movement map: It highlights the trajectory of the moving LSS UAV and minimizes the salt-and-pepper effect (Figure 10); also, the computation time is comparable with the wavelet transform method. For such applications (small moving drones/objects), this method represents a strong candidate to the classical approaches. 

## 5. Conclusions

This paper presents the actual context of the LSS UAV presence detection and characterization, and proposes a new alternative for their movement detection and characterization using advanced signal processing methods and the UWB sensing system.

The choice of the UWB sensing system is especially argued by the fact that this technology is able to detect small-dimension moving targets built from heterogeneous materials. Therefore, it surpasses the LoS limitations that the visual sensing system have and it does not interfere with other existing communication technologies. 

With a perpendicular positioning of the longitudinal axes of the UWB sensors and the use of several signal processing methods, our approach is able to detect and characterize the movement of a LSS UAV. 

The experiment presented in this paper emphasizes the fact that our approach detects indoor LSS UAVs intrusions, which represent a potential threat for classified or public areas with a special status. For example, given the audio/video recording capabilities of today’s LSS UAVs, possible specific use cases could be related to the unauthorized surveillance in areas such as: Window governmental office intrusion or balcony/window private building intrusion. Another possible application is the use of the LSS UAV in the context of “internet of everything” (IoE) and our proposed sensing system can be explored for the LSS UAV presence alert to the existing users [47].

The correlation method provides fast results, but a low contrast between the movement trajectory and static sensed objects. The spectrogram movement map provides better results for the LSS UAV trajectory, but it requires higher computation time and the trajectory quality is limited by the time-frequency resolution of the method.

The envelope detection provides better significant results than the latter two mentioned methods, pointing out the movement map trajectory in a clearer manner. The second advantage of this method is the reduced computation time/resources required.

With similar results as the envelope detection method, the wavelet transform approach is able to highlight the movement trajectory of the LSS UAV. The limitation of this method is the appropriate choice of the mother wavelet function and the required higher computation time/resources (but still usable for such applications).

The TDR RPA method provides the best results for the movement trajectory representation, clearly highlighting it and, by definition, filtering the salt-and-pepper noise. The proposed quantification measure, TDR, has the advantage to augment the sudden changes in the phase space and to minimize the noise effect. For our application, these sudden changes represent exactly the UWB pulses corresponding to the object’s movement. Although the required computation time is comparable with the wavelet transform method, processing is performed fast enough in order to trigger an alarm in real time for the surveyed system. Therefore, this drawback can be ignored, considering that the TDR RPA method emphasizes the trajectory of the LSS UAV the most. 

Therefore, our work describes a new approach for the LSS UAV indoor/office intrusion detection and tracking, proposing the UWB sensing system, and analyzing the results with advanced signal processing methods. Moreover, we discuss the trade-off between the movement map quality and computation time, which may be of interest depending on the application. 

The limitations of our approach are related to the UWB technology power standardization which makes it difficult to sense an object on long ranges. However, it is our belief that this approach can provide a good start for indoor LSS UAV detection and tracking.

Future developments of our work foresee the use of the presented approach on an automatic UWB sensing system based on machine learning methods, that is capable of discriminating between LSS UAVs and other moving targets.

## Figures and Tables

**Table 1:** Method performances.

Criterion	Envelope Detection ^1^	Wavelet Transform	TDR RPA
Movement map accuracy	-	0.83%	4.5%
Computation time [s]	0.2	2	2.4

^1^ Considered as reference [46].

## Appendix A

This appendix presents the computation algorithm for the signal-to-noise ratio discussed in Section 4.3.

Considering the signal for computation, s[n], n=1,2,…,L, then the signal-to-noise ratio is:

$$SNR=10\cdot \log_{10}\left(\frac{\sum_{i=M}^{N}{\left(s[i]\right)}^{2}}{N-M}\right)\;[dB]$$

where N,M are the sample limits for the interest region from the signal, 1≤M<N≤L and L is the length of the signal.
